# Synthesis of Dense 1,2,3-Triazole Polymers Soluble in Common Organic Solvents

**DOI:** 10.3390/polym13101627

**Published:** 2021-05-17

**Authors:** Shota Yamasaki, Yuri Kamon, Linlin Xu, Akihito Hashidzume

**Affiliations:** Department of Macromolecular Science, Graduate School of Science, Osaka University, 1-1 Machikaneyama-cho, Toyonaka, Osaka 560-0043, Japan; osakashogi@gmail.com (S.Y.); kamon@chem.sci.osaka-u.ac.jp (Y.K.); xul18@chem.sci.osaka-u.ac.jp (L.X.)

**Keywords:** Huisgen cycloaddition polymerization, copper(I)-catalyzed azide–alkyne cycloaddition polymerization, *t*-butyl 3-azide-6-hexynoate, 1,2,3-triazole

## Abstract

Aiming at synthesis of dense 1,2,3-triazole polymers soluble in common organic solvents, a new 3-azido-1-propyne derivative, i.e., *t*-butyl 4-azido-5-hexynoate (tBuAH), was synthesized and polymerized by copper(I)-catalyzed azide–alkyne cycloaddition (CuAAC) and Huisgen cycloaddition (HC). CuAAC polymerization produced poly(tBuAH) composed of 1,4-disubstituted 1,2,3-triazole units (1,4-units), whereas HC polymerization gave poly(tBuAH) composed of 1,4- and 1,5-disubstituted 1,2,3-triazole units (1,4- and 1,5-units). In HC polymerization, the fraction of 1,4-unit (*f*_1,4_) decreased with the permittivity of solvent used. Differential scanning calorimetry data indicated that the melting point of poly(tBuAH) increased from 61 to 89 °C with increasing *f*_1,4_ from 0.38 to 1.0, indicative of higher crystallinity of poly(tBuAH) composed of 1,4-unit. Preliminary steady-state fluorescence study indicated that all the poly(tBuAH) samples of different *f*_1,4_ emitted weak but significant fluorescence in DMF. The maximum of fluorescence band shifted from ca. 350 to ca. 450 nm with varying the excitation wavelength from 300 to 400 nm.

## 1. Introduction

Huisgen cycloaddition (HC) is a 1,3-dipolar cycloaddition, which gives 1,2,3-triazoles from azides and alkynes [1,2]. In a few decades, it is known that HC is catalyzed by copper(I) compounds to yield 1,4-disubstituted 1,2,3-triazoles efficiently and selectively under mild conditions [3,4,5,6]. This reaction has been widely known as copper(I)-catalyzed azide–alkyne cycloaddition (CuAAC). 1,2,3-Triazole is an aromatic five-membered ring composed of three nitrogen and two carbon atoms [7,8,9]. However, 1,2,3-triazole has a large dipole moment, and acts as a hydrogen bonding acceptor and a ligand for metal ions [10,11,12,13,14,15,16]. In some specific cases, 1,2,3-triazole also works as a hydrogen bonding donor [17,18,19,20,21,22,23]. On the basis of these features of 1,2,3-triazoles, polymers possessing 1,2,3-triazole units in their backbone are promising as new functional polymers. 1,2,3-Triazole polymers can be synthesized from monomers carrying azide and alkyne moieties or from a combination of diazides and dialkynes by HC or CuAAC [24,25,26,27,28,29,30,31,32].

We have been working on synthesis of dense 1,2,3-triazole polymers, and thus focusing of HC or CuAAC polymerization of 3-azido-1-propyne (AP) derivatives that possess azide and alkyne moieties connected through a carbon atom. Previously, we carried out CuAAC polymerization of the simplest AP derivatives, i.e., AP and 3-azido-1-butyne, using the corresponding bromides as monomer precursors, to obtain oligomers [33]. The oligomers were soluble in strong acids, e.g., concentrated sulfuric acid, nitric acid, and hydrochloric acid, whereas insoluble in all the common solvents examined. Because of the poor solubility, it was not possible to obtain high-molecular-weight polymers from the AP derivatives by CuAAC polymerization. However, it was possible to obtain dense 1,2,3-triazole oligomers or polymers soluble in common solvents by quaternization of the AP oligomer with methyl iodide [34] or by connecting to poly(ethylene glycol) (PEG) blocks, i.e., the formation of block copolymers [35,36]. The block copolymers of PEG and dense 1,2,3-triazole blocks form aggregates in water, e.g., spherical micelles and vesicles, depending on the ratio of block lengths, and undergo phase separation at higher temperatures because of cooperative dehydration of the PEG block [36]. In this study, we have synthesized an AP derivative possessing *t*-butyl ester, i.e., *t*-butyl 4-azido-5-hexynoate (tBuAH), which may improve the solubility of its polymer, and conducted CuAAC and HC polymerizations of tBuAH to obtain high-molecular-weight polymers soluble in common organic solvents. 

## 2. Materials and Methods

### 2.1. Measurements

^1^H and ^13^C NMR measurements were carried out on a JEOL JNM ECS400 or ECA500 spectrometer at 25 °C using chloroform-*d* (CDCl_3_) or dimethyl sulfoxide-*d*_6_ (DMSO-*d*_6_) as a solvent. Chemical shifts were referenced to the signal due to tetramethylsilane (TMS) (0 ppm). Heteronuclear single quantum correlation (HSQC) and heteronuclear multiple bond correlation (HMBC) data were recorded on a Bruker AVANCE 700 NMR spectrometer at 25 °C using DMSO-*d*_6_ as a solvent. Chemical shifts were referenced to the signals due to the solvent. High-resolution mass spectra (HRMS) were measured on a Thermo Fisher Scientific Orbitrap XL using an electrospray ionization (ESI) source. Methanol was used as a solvent. Internal calibration of ESI-MS was carried out using the monoisotopic peaks of sodium adduct ion of diethyl phthalate (*m*/*z* 314.1410), protonated ion of di-2-ethylhexyl phthalate (*m*/*z* 391.2843), and sodium adduct ion of di-2-ethylhexyl phthalate (*m*/*z* 413.2662). Size exclusion chromatography (SEC) measurements were carried out at 25 °C on a TOSOH HLC-8320GPC system with TOSOH TSK columns SuperAWM-H using dimethyl sulfoxide (DMSO) containing lithium bromide (1.05 g L^−1^) as the eluent at a flow rate of 0.4 mL min^−1^. The molecular weights were calibrated with standard samples of poly(ethylene glycol) (PEG) and poly(ethylene oxide) (PEO) (Scientific Polymer Products, Inc. (Ontario, NY, USA). Sample solutions were filtrated with a DISMIC-13JP PTFE 0.50 μm filter just prior to injection. SEC experiments were also performed on a system composed of a Viscotek VE 1122 pump, two TOSOH TSKgel GMH_HR_-M columns connected in series, and a Viscotek TDA 305 triple detector, which monitored signals by refractive index, laser light scattering (*λ* = 670 nm, *θ* = 7° and 90°, where *λ* and *θ* denote the wavelength of incident light and scattering angle, respectively), and differential pressure viscometer, using THF as an eluent at 40 °C. The molecular weights were determined using the light scattering signals along with the values of specific refractive index increment determined on an Otsuka Electronics DRM-3000 differential refractometer. Sample solutions were filtrated with a DISMIC-13JP PTFE 0.50 μm filter (ADVANTEC Toyo Kaisha Ltd. (Tokyo, Japan)) just prior to injection. Elemental analyses were performed on a Yanaco CHN Corder MT-6 (Yanaco Apparatus Development Laboratory Co., Ltd. (Kyoto, Japan)). Differential scanning calorimetry (DSC) experiments were carried out with a DSC7020 differential scanning calorimeter (Hitachi High-Tech Science Corporation (Tokyo, Japan)) under a stream of nitrogen (200 mL min^−1^). The rates of heating and cooling processes were set to 10 °C min^−1^. DSC curves were recorded during three thermal scan cycles composed of heating, hold for 5 min, cooling, and hold for 5 min in a temperature range of −50 to 200 °C. The melting points (*T*_m_) were determined from the minimum of negative signal in the first heating scan. Using the second heating scans, the glass transition temperatures (*T*_g_) were estimated from the temperature at which the time derivative of the heat flow curve exhibited a peak corresponding to the inflection point (nearly equal to the midpoint) of the heat flow jump. Absorption spectra were collected on a JASCO V-650 spectrophotometer using a 1.0 cm path length quartz cuvette at 25 °C. Steady state fluorescence spectra were recorded on a HITACHI F-2500 spectrophotometer using a 1.0 cm path length quartz cuvette at 25 °C. The slit widths for both the excitation and emission sides were kept at 5.0 nm during measurement. 

### 2.2. Materials

Tetrahydrofuran (THF), phosphorus tribromide (PBr_3_), triethylamine, triphenylphosphine (PPh_3_), copper(II) sulfate pentahydrate (CuSO_4_•5H_2_O), sodium azide (NaN_3_), and ethylenediaminetetraacetic acid (EDTA) were purchased from FUJIFILM Wako Pure Chemical Corp. (Osaka, Japan). *t*-Butyl alcohol, 1-(3-dimethylaminopropyl)-3-ethylcarbodiimide hydrochloride (EDC), *t*-butyldimethylsilylacetylene, diphenylphosphoryl azide (DPPA), diisopropyl azodicarboxylate (DIAD), and tetrabutylammonium fluoride (TBAF) were purchased from Tokyo Chemical Industry Co. Ltd. (Tokyo, Japan). A solution of butyllithium (BuLi) in hexane (1.55 M) was purchased from Kanto Chemical Co., Inc. (Tokyo, Japan). Succinic anhydride, *N*-hydroxysuccinimide (NHS), *N*,*N*-dimethyl-4-aminopyridine (DMAP), *N*,*O*-dimethylhydroxylamine hydrochloride, *N*,*N*-diisopropylethylamine (DIPEA), 1-hydroxybenzotriazole (HOBt), acetic acid (AcOH), copper(I) bromide (CuBr), copper(I) iodide (CuI), L-(+)-ascorbic acid sodium salt (NaAsc), and sodium borohydride (NaBH_4_) were purchased from Nacalai Tesque, Inc. (Kyoto, Japan). For thin layer chromatography (TLC) analysis throughout this work, Merck precoated TLC plates (silica gel 60 F254) were used. The products were purified by column chromatography using silica gel 60 (Nacalai Tesque, spherical, neutrality). 

*t*-Butyl 4-(methoxy(methyl)amino)-4-oxobutanoate (**3**) was prepared using succinic anhydride as the starting material according to the procedure of Falciani et al. [37]. 

### 2.3. Synthesis of t-Butyl 4-Azido-5-Hexynoate (tBuAH)

#### 2.3.1. Synthesis of *t*-Butyl 6-(*t*-Butyldimethylsilyl)-4-Oxo-5-Hexynoate (**4**) 

A solution of BuLi in hexane (1.55 M, 8.00 mL, 12.4 mmol) was added dropwise to a schlenk flask containing a solution of *t*-butyldimethylsilylacetylene (2.26 mL, 12.4 mmol) in dry THF (25 mL) at −78 °C using a solid CO_2_-methanol bath under a nitrogen atmosphere. Then, the reaction solution was stirred for 1 h at −78 °C under a nitrogen atmosphere. A solution of **3** (2.69 g, 12.4 mmol) in dry THF (4 mL) was added dropwise to the reaction solution at −78 °C under a nitrogen atmosphere. Then, the reaction solution was stirred for 14 h at −78 °C under a nitrogen atmosphere. After the reaction mixture was poured into 1 M HCl (100 mL), the product was extracted with ethyl acetate (3 × 40 mL). The organic phases were combined, and the combined organic layer was washed with saturated NaCl (100 mL). The organic layer was dried with anhydrous Na_2_SO_4_. After evaporation of the solvent, the product **4** was purified by column chromatography using a mixed solvent of hexane and ethyl acetate (20/1–4/1, *v/v*) as eluent to obtain colorless oil (2.99 g, 81.4% yield). ^1^H NMR (400 MHz, CDCl_3_) *δ* (ppm): 2.85 (t, *J* = 6.8 Hz, 2H, CH_2_), 2.57 (t, *J* = 6.8 Hz, 2H, CH_2_), 1.44 (s, 9H, *t*-C_4_H_9_), 0.97 (s, 9H, *t*-C_4_H_9_), 0.18 (s, 6H, CH_3_). ^13^C NMR (100 MHz, CDCl_3_) *δ* (ppm): 185.62, 171.39, 102.54, 97.35, 81.08, 40.40, 29.39, 28.22, 26.15, 16.72, 5.00. HRMS (ESI) *m*/*z*: calcd for C_16_H_28_NaO_3_Si [M + Na]^+^, 319.1705; found, 319.1754 [38]. 

#### 2.3.2. Synthesis of *t*-Butyl 6-(*t*-Butyldimethylsilyl)-4-Hydroxy-5-Hexynoate (**5**) 

NaBH_4_ (0.794 g, 21.0 mmol) was added to a two-neck flask containing **4** (2.49 g, 8.38 mmol) in dry methanol (30 mL) under a nitrogen atmosphere. After the reaction solution was stirred at room temperature for 9 h, 1 M HCl (120 mL) was added to the reaction solution. The product was then extracted with ethyl acetate (3 × 30 mL). The organic phases were combined, and the combined organic layer was washed with saturated NaCl (80 mL). The organic phase was dried with Na_2_SO_4_. After evaporation of the solvent, the product **5** was purified by column chromatography using a mixed solvent of hexane and ethyl acetate (40/1–5/1, *v/v*) as eluent to obtain colorless oil (2.25 g, 89.8% yield). ^1^H NMR (400 MHz, CDCl_3_) *δ* (ppm): 4.46 (q, *J* = 6.0 Hz, 1H, CH), 2.64 (d, *J* = 5.8 Hz, 1H, OH), 2.56–2.39 (m, 2H, CH_2_), 1.98 (m, 2H, CH_2_), 1.45 (s, 9H, *t*-C_4_H_9_), 0.93 (s, 9H, *t*-C_4_H_9_), 0.11 (s, 6H, CH_3_). ^13^C NMR (100 MHz, CDCl_3_) *δ* (ppm): 173.32, 106.86, 88.12, 80.83, 62.12, 32.80, 31.52, 28.24, 26.21, 16.60, 4.50. HRMS (ESI) *m*/*z*: calcd for C_16_H_30_NaO_3_Si [M + Na]^+^, 321.1862; found: 321.1918 [38]. 

#### 2.3.3. Synthesis of *t*-Butyl 4-Azido-6-(*t*-Butyldimethylsilyl)-5-Hexynoate (**6**) 

PPh_3_ (1.60 g, 6.10 mmol), DIAD (1.20 mL, 6.11 mmol), and DPPA (1.32 mL, 6.13 mmol) were added to a solution of **5** (1.55 g, 5.19 mmol) in dry THF (20 mL) at 0 °C under a nitrogen atmosphere. The reaction solution was allowed to warm to room temperature with stirring for 24 h. After evaporation of the solvent, the product was purified by column chromatography using a mixed solvent of hexane and ethyl acetate (40/1, *v/v*) as eluent. The product **6** was obtained as colorless oil (1.31 g, 78.0% yield). ^1^H NMR (400 MHz, CDCl_3_) *δ* (ppm): 4.21 (t, *J* = 6.8 Hz, 1H, CH), 2.40 (t, *J* = 7.2 Hz, 2H, CH_2_), 1.96 (m, 2H, CH_2_), 1.45 (s, 9H, *t*-C_4_H_9_), 0.95 (s, 9H, *t*-C_4_H_9_), 0.14 (s, 6H, CH_3_). ^13^C NMR (CDCl_3_, 100 MHz) *δ* (ppm): 171.7, 100.3, 91.1, 80.6, 52.7, 31.3, 30.5, 28.0, 25.9, 16.3, −4.7. HRMS (ESI) *m*/*z*: calcd for C_16_H_29_N_3_NaO_2_Si [M + Na]^+^, 346.1972; found, 346.2041 [39]. 

#### 2.3.4. Synthesis of *t*-Butyl 4-Azido-5-Hexynoate (tBuAH) 

AcOH (0.50 mL, 8.7 mmol) and a solution of TBAF in THF (1M, 8.7 mL, 8.7 mmol) were added to a solution of **6** (1.37 g, 4.25 mmol) in dry THF (15 mL) at 0 °C under a nitrogen atmosphere. After the reaction solution was allowed to warm to room temperature with stirring for 14 h, the reaction solution was poured into 10% citric acid (60 mL). Then, the product was extracted with a mixed solvent of hexane and ethyl acetate (4/1, *v/v*) (3 × 30 mL). The organic phases were combined, and the combined organic layer was washed with saturated NaCl (60 mL). The organic phase was dried with anhydrous Na_2_SO_4_. After evaporation of the solvent, the product was purified by column chromatography using a mixed solvent of hexane and ethyl acetate (40/1–10/1, *v/v*) as eluent to obtain colorless oil (0.664 g, 3.17 mmol, 74.6%). ^1^H NMR (CDCl_3_, 400 MHz) *δ* (ppm): 4.21 (td, *J* = 6.8 Hz, 2.3 Hz, 1H, CH), 2.59 (d, *J* = 2.3 Hz, 1H, C≡CH), 2.41 (m, 2H, CH_2_), 1.98 (m, 2H, CH_2_), 1.45 (s, 9H, *t*-C_4_H_9_). ^13^C NMR (CDCl_3_, 100 MHz) *δ* (ppm): 171.7, 100.3, 91.1, 80.6, 52.7, 31.3, 30.5, 28.0, 25.9, 16.3, -4.7. HRMS (ESI) *m*/*z*: calcd for C_10_H_15_N_3_NaO_2_ [2M + Na]^+^, 441.2226; found, 441.2220 [40]. 

### 2.4. Polymerization of tBuAH

#### 2.4.1. Copper(I)-Catalyzed Azide–Alkyne Cycloaddition (CuAAC) Polymerization of tBuAH

A typical procedure of CuAAC polymerization is described below. 

A copper(I) catalyst (0.11 mmol) and NaAsc (65 mg, 0.33 mmol) were added to a solution of tBuAH (0.225 g, 1.08 mmol) in a solvent (1.0 mL) under a nitrogen atmosphere. The reaction mixture was warmed using an oil bath thermostated at 60 °C with stirring for 48 h. After the reaction mixture was cooled down to room temperature, ethyl acetate (10 mL) was added to the mixture. The organic layer was washed with water (10 mL) and with 0.50 M EDTA (2 × 10 mL). The polymer obtained was recovered by reprecipitation with hexane (30 mL). The polymer was dried at 40 °C under reduced pressure. ^1^H NMR (CDCl_3_, 400 MHz) *δ* (ppm): 8.4 (1H, 1,2,3-triazole CH), 6.0 (1H, CH), 2.5 (2H, CH_2_), 2.1 (2H, CH_2_), 1.4 (9H, *t*-C_4_H_9_). Anal. calcd for (C_10_H_15_N_3_O_2_)(CuO)_0.13_: C, 54.70; H, 6.89; N, 19.13. Found: C, 55.12; H, 6.90; N, 18.78. 

#### 2.4.2. Huisgen Cycloaddition (HC) Polymerization of tBuAH

A typical procedure of HC polymerization is described below. 

A solution of tBuAH (0.225 g, 1.08 mmol) in a solvent (1.0 mL) was warmed using an oil bath thermostated at 60 °C with stirring for 48 h under a nitrogen atmosphere. After the reaction mixture was cooled down to room temperature, ethyl acetate (10 mL) was added to the mixture. The organic layer was washed with water (10 mL). The polymer obtained was recovered by reprecipitation with hexane (30 mL). The polymer was dried at 40 °C under reduced pressure. ^1^H NMR (CDCl_3_, 400 MHz) *δ* (ppm): 8.4–7.6 (1H, 1,2,3-triazole CH), 6.3–5.8 (1H, CH), 2.5 (2H, CH_2_), 2.1 (2H, CH_2_), 1.4 (9H, *t*-C_4_H_9_). Anal. calcd for C_10_H_15_N_3_O_2_: C, 57.40; H, 7.23; N, 20.08. Found: C, 56.86; H, 7.23; N, 19.87. 

### 2.5. Density Functional Theory (DFT) and Time-Dependent DFT (TDDFT) Calculations

DFT and DTDFT calculations were carried out for 1,4-dimethyl-1,2,3-triazole and 1,5-dimethyl-1,2,3-triazole using the Gaussian 09 program [41] to estimate the dipole moments, and the highest-occupied and lowest-unoccupied molecular orbitals (HOMO and LUMO, respectively). In all the calculations, DFT with B3LYP functional was used, and 6-31 + G (d) basis sets were applied for the hydrogen, carbon, and nitrogen atoms. All the geometries were fully optimized. 

## 3. Results and Discussion

**Scheme 1:**
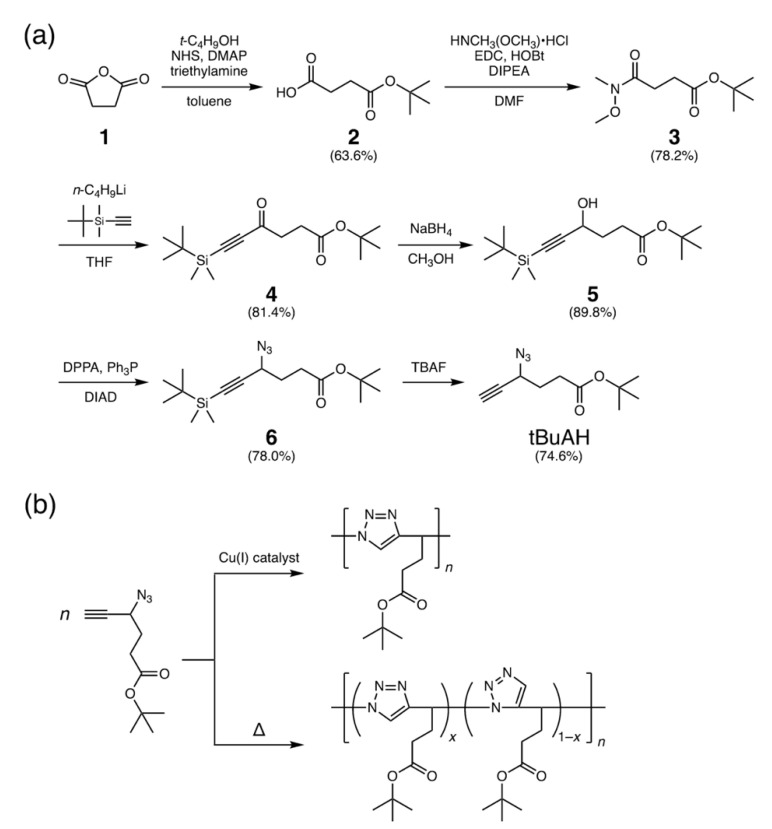
Synthesis (**a**) and polymerization of tBuAH (**b**).

**Figure 1:**
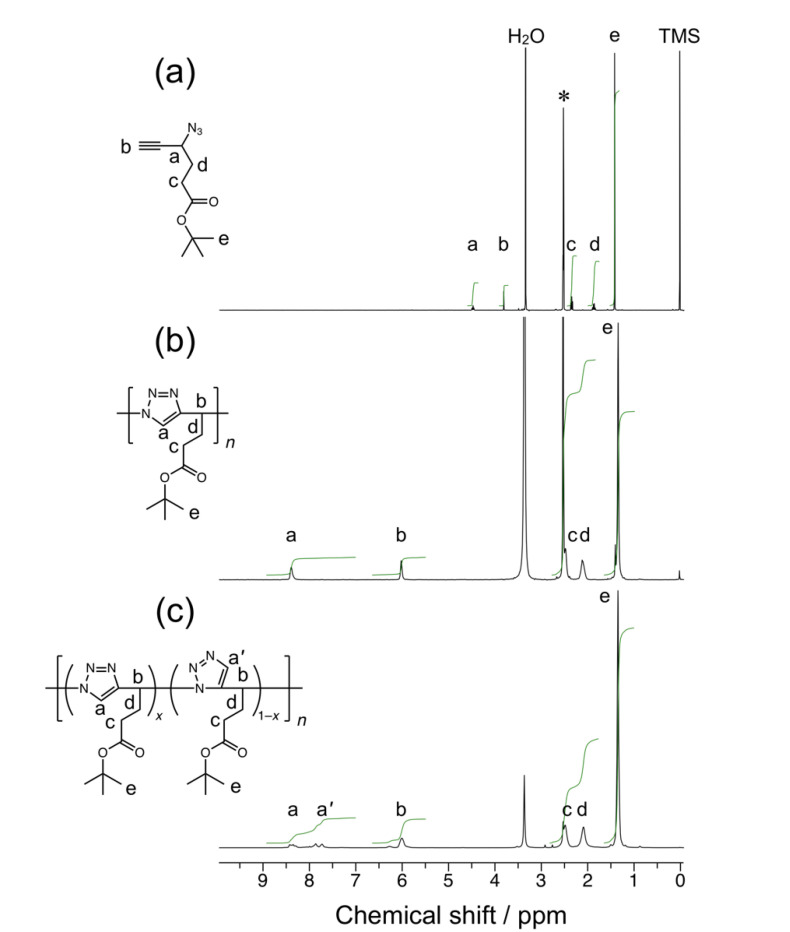
^1^H NMR spectra for tBuAH (**a**) and poly(tBuAH) obtained by CuAAC polymerization (**b**) and HC polymerization (DMSO-*d*_6_) (**c**). The black and green curves are spectra and integrals, respectively.

After some trials, we have designed a 5-hexynoic acid derivative, *t*-butyl 4-azido-5-hexynoate (tBuAH), possessing a *t*-butyl ester moiety through two methylene carbons. According to Scheme 1a, we have synthesized tBuAH using succinic anhydride as the starting material. Succinic anhydride was opened with *t*-butyl alcohol using a coupling agent, *N*-hydroxysuccinimide (NHS), to obtain mono-*t*-butyl succinate (**2**). The remaining carboxylic acid moiety was coupled with *N*,*O*-dimethylhydroxylamine in the presence of 1-(3-dimethylaminopropyl)-3-ethylcarbodiimide hydrochloride (EDC), 1-hydroxybenzotriazole (HOBt), and *N*,*N*-diisopropylethylamine (DIPEA) to form a Weinreb amide (**3**). The Weinreb amide **3** was dealt with *t*-butyldimethylacetylene lithiated using butyllithium (BuLi) to introduce an ethynyl moiety carrying a *t*-butyldimethylsilyl (TBDMS) protecting group. The carbonyl of **4** on the ethynyl side was reduced to hydroxy group with sodium borohydride (NaBH_4_). The hydroxy group of **5** was replaced with an azide group using diphenylphosphoryl azide (DPPA). After removal of the TBDMS protecting group of **6**, the monomer, tBuAH, was obtained. Each reaction proceeded in a reasonable yield, and all the compounds were characterized by ^1^H and ^13^C NMR, and MS (see Materials and Methods, and Figures S1–S6 in the Supplementary Materials). Figure 1a displays a typical example of ^1^H NMR spectra for tBuAH. The spectrum contains signals ascribable to the ethynyl and methine protons at 2.6 and 4.2 ppm, respectively. There are signals assignable to the methylene protons at 2.4 and 2.0 ppm. The spectrum also exhibits an intense singlet signal due to the *t*-butyl group at 1.4 ppm. The ratio of area intensities agrees well with the structure of tBuAH. ^13^C NMR and MS data also supported the formation of tBuAH (see Materials and Methods and Figures S7 and S8 in the Supplementary Materials). These characterization data led us to conclude that tBuAH was successfully synthesized. 

Table 1 summarizes typical examples of conditions and results of CuAAC and HC polymerization of tBuAH. CuAAC polymerization of tBuAH was carried out in *N*,*N*-dimethylformamide (DMF) at 60 °C for 48 h, using copper(I) bromide (CuBr), copper(I) iodide (CuI), and a pair of copper(II) sulfate pentahydrate and L-(+)-ascorbic acid sodium salt (CuSO_4_•5H_2_O/NaAsc) as copper(I) catalyst (runs 1–3 in Table 1). The polymer obtained was purified by washing with water and 0.50 M EDTA, followed by reprecipitation using a pair of ethyl acetate and hexane as good and poor solvents, respectively. The polymer yield increased in the order of CuI < CuSO_4_•5H_2_O/NaAsc < CuBr, indicating that CuBr is the most efficient catalyst under the conditions used in this study. For all the three copper(I) catalysts, poly(tBuAH) samples with PEG-calibrated *M*_w_ > 8 × 10^3^ were obtained. The *M*_w_/*M*_n_ ranges 1.4–1.6. The absolute *M*_w_ was determined to be 1.6 × 10^4^ for poly(tBuAH) obtained with CuSO_4_•5H_2_O/NaAsc (run 4 in Table 1) using the light scattering data obtained with a size exclusion chromatography (SEC) system equipped with a triple detector. These observations are indicative of the successful formation of high-molecular-weight poly(tBuAH). It is noteworthy that tBuAH is readily polymerized without any copper(I) catalyst by HC polymerization. Table 1 also contains typical examples of the conditions and results of HC polymerization of tBuAH in three different solvents under copper(I)-free conditions (runs 5–7). As can be seen in the table, HC polymerization also produced poly(tBuAH) of high *M*_w_ = (2.6 − 8.0) × 10^4^. It should be noted here that the polymer yields for HC were higher than that for CuAAC, indicating that copper(I) ions somehow retards cycloaddition. This may be because 1,2,3-triazole units in poly(tBuAH) coordinate copper(I) ions to form complexes, in which steric hindrance may retard CuAAC. 

**Figure 2:**
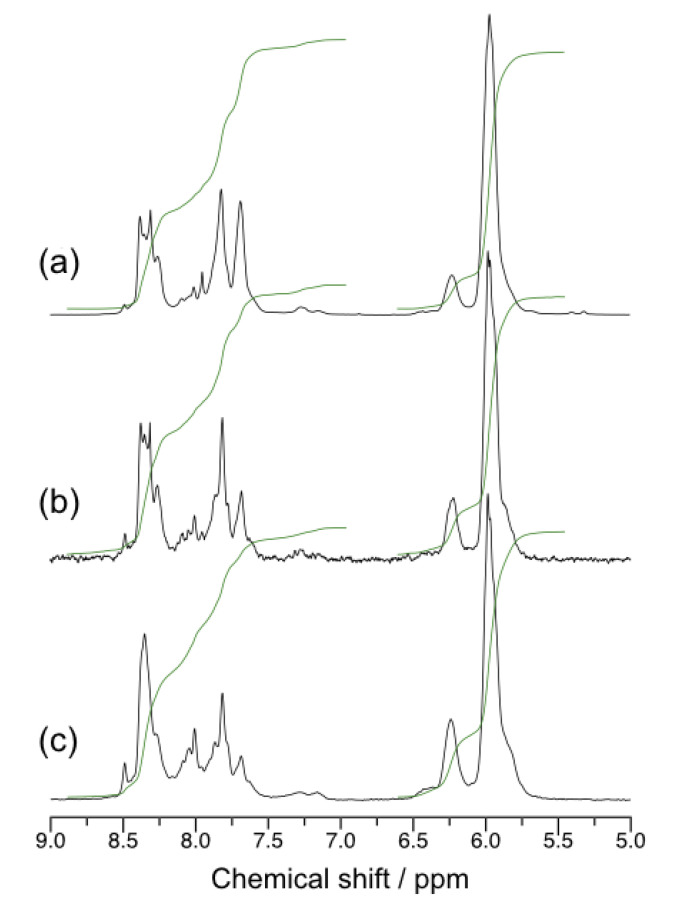
Expanded ^1^H NMR for poly(tBuAH) samples obtained by HC polymerization using DMF (**a**), THF (**b**), and toluene as solvent (DMSO-*d*_6_) (**c**). The black and green curves are spectra and integrals, respectively.

The structure of poly(tBuAH) obtained was characterized by elemental analysis and ^1^H NMR. As can be seen in Materials and Methods, the C, H, and N contents of polymers agreed well with those of tBuAH, indicating that the polymers were obtained by CuAAC and HC. It should be noted here that a poly(tBuAH) sample obtained by CuAAC polymerization contained a significant amount of inorganic salt, which may be derived from the copper catalyst utilized. Since the polymer sample was washed with an aqueous solution of EDTA, it is likely that poly(tBuAH) captures strongly copper ions [33]. Figure 1b,c exhibit ^1^H NMR spectra for the polymers obtained by CuAAC and HC, respectively. Figure 1b shows rather sharp signals assignable to the 1,2,3-triazole and methine protons at 8.4 and 6.0 ppm, respectively. This spectrum also contains signals due to the methylene protons at 2.5 and 2.1 ppm as well as a signal of *t*-butyl protons at 1.4 ppm. The ratio of area intensities agrees well with the polymer structure shown in Scheme 1b. These observations indicate that CuAAC polymerization of tBuAH yields poly(tBuAH) composed of 1,4-disubstituted 1,2,3-triazole units (1,4-units). On the other hand, the ^1^H NMR spectrum (Figure 1c) for the poly(tBuAH) obtained by HC polymerization exhibits more complicated signals ascribable to the triazole (7.6–8.4 ppm) and methine protons (5.8–6.4 ppm) because of 1,4- and 1,5-disubstituted 1,2,3-triazole units (1,4- and 1,5-units) (Scheme 1b). It should be noted here that the triazole and methine protons exhibit separate signals based on the triads and diads, respectively, as can be seen in Figure S9 in the Supplementary Materials. On the basis of the HSQC for poly(tBuAH) (run 6) (Figure S10 in the Supplementary Materials), the signals in the regions of 8.2–8.4 and 7.6–8.2 ppm are assignable to the 1,4- and 1,5-units, respectively. The HMBC data for poly(tBuAH) (run 6) confirmed that the signals at ca. 6.0 and 6.2 ppm were ascribable to the methine protons of 1,4/1,4-, 1,4/1,5-, 1,5/1,4-diads and 1,5/1,5-diad, respectively (Figure S11 in the Supplementary Materials) [42]. From the ratios of area intensities of triazole protons (Figure 2), the fractions of 1,4-unit (*f*_1,4_ (= *x* in Scheme 1)) were evaluated, as summarized in Table 1. The fraction, *f*_1,4_, decreases from 0.49 to 0.38 with increasing the permittivity of solvent used. This may be because the 1,5-disubstituted 1,2,3-triazole possesses a larger dipole moment than that of the 1,4-disubstituted 1,2,3-triazole (Figure S12 in the Supplementary Materials). 

**Figure 3:**
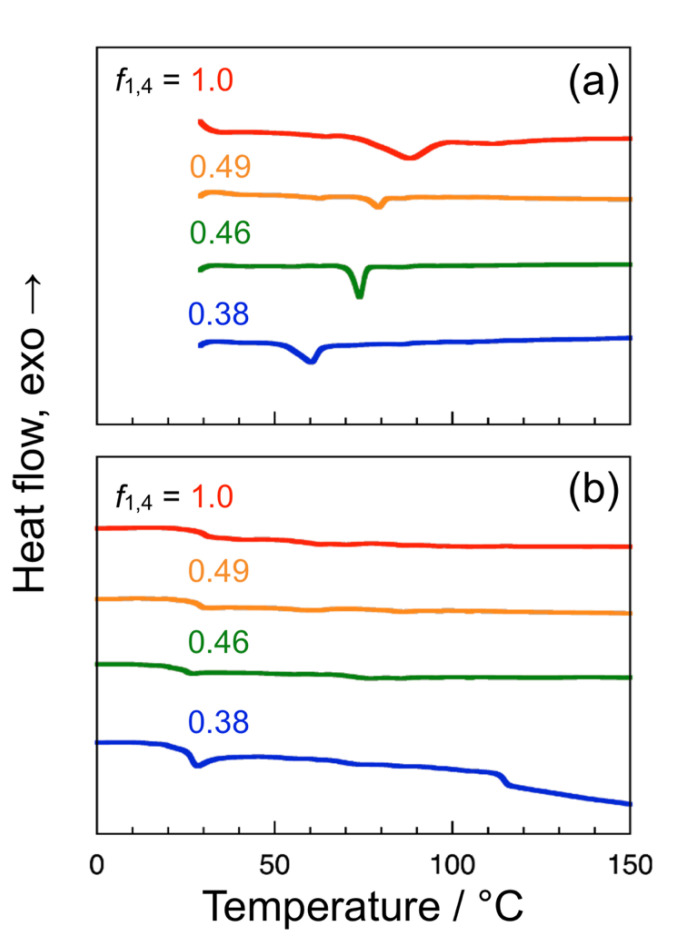
DSC data for the poly(tBuAH) samples of different *f*_1,4_ under a stream of nitrogen (200 mL min^−1^); the first (**a**) and second heating scans (**b**). The heating rate was set at 10 °C min^−1^.

Thermal properties of the poly(tBuAH) samples obtained by CuAAC and HC were investigated by differential scanning calorimetry (DSC). Figure 3 displays a typical example of DSC data obtained. As can be seen in Figure 3a, the first heating scans for all the poly(tBuAH) samples of *f*_1,4_ = 1.0, 0.48, 0.46, and 0.38 contain an endothermic signal due to the melting of poly(tBuAH). The melting points (*T*_m_) were determined from the minimum of endothermic signal (Table 2). It should be noted here that *T*_m_ increases from 61 to 89 °C with increasing *f*_1,4_ from 0.38 to 1.0. The heat of fusion for the poly(tBuAH) sample composed of 1,4-units (21.4 J g^−1^) is also larger than those for the poly(tBuAH) samples composed of 1,4- and 1,5-units (4.9–6.5 J g^−1^). These observations indicate that the poly(tBuAH) sample composed of 1,4-units shows higher crystallinity presumably because of the regular structure. From the second heating scans (Figure 3b), the glass transition temperatures (*T*_g_) were roughly estimated to be 22–30 °C for the poly(tBuAH) samples (Table 2). (The DSC curve for poly(tBuAH) of *f*_1,4_ = 0.38 seems to show the second glass transition at ca. 114 °C presumably because of a larger fraction of 1,5/1,5-diad, but we are not sure about details of the transition.) 

As summarized in Table 3, the poly(tBuAH) samples obtained were well soluble in polar organic solvents, e.g., DMSO, DMF, methanol, THF, and ethyl acetate, and in halogenated solvents, e.g., chloroform and dichloromethane. The solubilities of poly(tBuAH) samples obtained by CuAAC and HC were almost the same. These data indicate that the *t*-butyl ester group markedly improves the solubility of polymers of AP derivatives, resulting in the formation of higher molecular weight polymers. 

**Figure 4:**
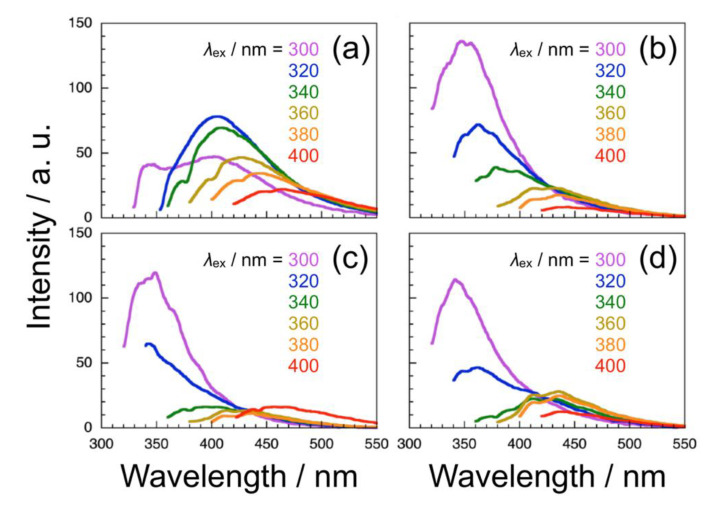
Steady-state fluorescence spectra for 1.0 g L^−1^ solutions of the poly(tBuAH) samples of different *f*_1,4_ in DMF obtained by excitation at varying wavelengths from 300 to 400 nm; *f*_1,4_ = 1.0 (**a**), 0.49 (**b**), 0.46 (**c**), and 0.38 (**d**).

Our previous work on diblock copolymers of PEG and AP blocks demonstrated that the dense 1,2,3-triazole block emitted weak fluorescence in the molecularly dispersed state [35]. Thus, the photophysical behavior of solutions of the poly(tBuAH) samples in DMF was preliminarily examined by steady-state fluorescence spectroscopy because fluorescent polymers may be applicable to some fields, e.g., energy conversion and sensing. Figure 4 compares steady-state fluorescence spectra for 1.0 g L^−1^ DMF solutions of the four poly(tBuAH) samples of *f*_1,4_ = 1.0, 0.49, 0.46, and 0.38 with excitation at different wavelengths (300–400 nm). These spectra exhibit that all the sample solutions emit weak but significant fluorescence. The maximum of fluorescence band shifts from ca. 350 to ca. 450 nm with varying the excitation wavelength from 300 to 400 nm. In the cases of the poly(tBuAH) samples composed of 1,4- and 1,5-units (i.e., *f*_1,4_ = 0.49, 0.46, and 0.38), the fluorescence intensity decreases with increasing the excitation wavelength. On the other hand, the poly(tBuAH) sample of 1,4-unit (i.e., *f*_1,4_ = 1.0) emits less intense fluorescence with excitation at 300 nm. It should be noted here that the fluorescence intensities for the sample of *f*_1,4_ = 1.0 with excitation at wavelengths ≥340 nm were higher than those for the other samples (*f*_1,4_ = 0.49, 0.46, and 0.38). DFT and TDDFT calculations have indicated that 1,5-dimethyl-1,2,3-triazole exhibits adsorption and emission maxima shorter than those for 1,4-dimethyl-1,2,3-triazole (Figure S13 and Table S1 in the Supplementary Materials). It is thus likely that these differences in the fluorescence behavior for poly(tBuAH) samples are ascribable to the polymer isomeric structures. 

## 4. Conclusions

We have synthesized tBuAH and conducted its CuAAC and HC polymerizations in this study, aiming at the formation of dense 1,2,3-triazole polymers soluble in common organic solvents. CuAAC polymerization of tBuAH using CuSO_4_•5H_2_O/NaAsc in DMSO at 60 °C yielded poly(tBuAH) with *M*_w_ = 1.6 × 10^4^. HC polymerization of tBuAH proceeded to obtain polymer samples of high *M*_w_ (= (2.6 − 8.0) × 10^4^) in quantitative yield. The *t*-butyl ester side chain improved the solubility of polymer, resulting in the formation of high-molecular-weight poly(tBuAH). The structure of poly(tBuAH) samples was characterized by ^1^H NMR, indicating that CuAAC polymerization produced poly(tBuAH) composed of 1,4-units, whereas HC polymerization gave poly(tBuAH) composed of 1,4- and 1,5-units. In HC polymerization, *f*_1,4_ decreased with increasing the permittivity of the solvent used. Thermal properties of the poly(tBuAH) samples were investigated by DSC. All the polymer samples showed endothermic signals due to melting in the first heating scan, and exhibited signals ascribable to glass transition in the second heating scan. *T*_m_ increased from 61 to 89 °C with increasing *f*_1,4_ from 0.38 to 1.0, indicating that poly(tBuAH) composed of 1,4-unit has a higher crystallinity presumably because of the regular structure. *T*_g_ ranged 22–30 °C. The poly(tBuAH) samples emitted weak but significant fluorescence in DMF. The maximum of fluorescence band shifted from ca. 350 to ca. 450 nm with varying the excitation wavelength from 300 to 400 nm. In the cases of the poly(tBuAH) samples composed of 1,4- and 1,5-units, the fluorescence intensity decreased with increasing the excitation wavelength. On the other hand, the poly(tBuAH) sample of 1,4-unit emitted less intense fluorescence with excitation at 300 nm. 

## Figures and Tables

**Table 1:** Conditions and results of polymerization of tBuAH ^1^.

Run	Solvent	Cu(I) Catalyst	Yield/%	*M*_w_ ^2^/10^3^	*M*_w_/*M*_n_ ^2^	*M*_w_ ^3^/10^3^	*M*_w_/*M*_n_ ^3^	*f*_1,4_ ^4^
1	DMF	CuBr 5	83	9.1	1.4	-	-	1.0
2	DMF	CuI 5	26	8.3	1.6	-	-	1.0
3	DMF	CuSO4•5H2O/NaAsc	53	9.3	1.4	-	-	1.0
4	DMSO	CuSO4•5H2O/NaAsc	44	7.7	1.5	16	1.3	1.0
5	DMF	-	96	12	1.5	26	1.3	0.38
6	THF	-	94	46	2.2	80	1.8	0.46
7	toluene	-	89	45	2.4	78	1.6	0.49

^1^ At 60 °C for 48 h. Purified by washing with methanol, 0.50 M EDTA, and water. ^2^ Determined by SEC measurements at 25 °C using DMSO containing 1.05 g L^−1^ LiBr as eluent. Molecular weights were calibrated with PEG and PEO standard samples. ^3^ Determined using light scattering signals obtained by SEC measurements at 40 °C using THF as eluent. ^4^ The fraction of 1,4-unit determined by ^1^H NMR. ^5^ NaAsc (3 equivalent) was added as an additive.

**Table 2:** Thermal properties of poly(tBuAH) samples determined by DSC.

*f*_1,4_	*T*_m_ ^1^/°C	Heat of Fusion ^1^/J g^−1^	*T*_g_ ^2^/°C	*C*_p_ at *T*_g_ ^2^/J deg^−1^ g^−1^
1.0	89	21	30	1.1
0.49	79	4.9	24	0.71
0.46	74	6.5	22	0.51
0.38	64	5.6	28 114	0.98 0.57

^1^ Determined from the first heating scan of DSC. ^2^ Estimated from the second heating scan of DSC.

**Table 3:** Results of solubility test for poly(tBuAH) samples obtained by CuAAC and HC polymerizations.

Solvent	Solubility ^1^	
	CuAAC	HC ^2^
DMSO	++	++
DMF	++	++
THF	++	++
chloroform	++	++
dichloromethane	++	++
acetone	++	++
ethyl acetate	+	+
acetonitrile	+	+
methanol	–	–
water	–	–
toluene	–	–
diethyl ether	–	–

^1^ “++”, “+”, and “–” denote soluble at ≥10 g L^−1^, soluble at <10 g L^−1^, and insoluble, respectively. ^2^ Poly(tBuAH) sample of *f*_1,4_ = 0.38.

## Data Availability

The data presented in this study are available on request from the corresponding author.

## Supplementary Materials

The following are available online at https://www.mdpi.com/article/10.3390/polym13101627/s1, Figure S1: ^1^H NMR spectrum for *t*-butyl 6-(*t*-butyldimethylsily)-4-oxo-5-hexynoate (**4**) (CDCl_3_), Figure S2: ^13^C NMR spectrum for *t*-butyl 6-(*t*-butyldimethylsilyl)-4-oxo-5-hexynoate (**4**) (CDCl_3_), Figure S3: ^1^H NMR spectrum for *t*-butyl 6-(*t*-butyldimethylsilyl)-4-hydroxy-5-hexynoate (**5**) (CDCl_3_), Figure S4: ^13^C NMR spectrum for *t*-butyl 6-(*t*-butyldimethylsilyl)-4-hydroxy-5-hexynoate (**5**) (CDCl_3_), Figure S5: ^1^H NMR spectrum for *t*-butyl 4-azido-6-(t-butyldimethylsilyl)-5-hexynoate (**6**) (CDCl_3_), Figure S6: ^13^C NMR spectrum for *t*-butyl 4-azido-6-(*t*-butyldimethylsilyl)-5-hexynoate (**6**) (CDCl_3_), Figure S7: ^13^C NMR spectrum for tBuAH (CDCl_3_), Figure S8: ESI-MS for tBuAH, Figure S9: The structures of diads and triads for poly(tBuAH), Figure S10: HSQC charts for poly(tBuAH) composed of 1,4- and 1,5-units, Figure S11: HMBC charts for poly(tBuAH) composed of 1,4- and 1,5-units, Figure S12: DFT calculation data for 1,4-dimethyl-1,2,3-triazole and 1,5-dimethyl-1,2,3-triazole, Figure S13: Optimized structures, the lowest-unoccupied molecular orbitals (LUMO), and the highest-occupied molecular orbitals (HOMO) of 1,4-dimethyl-1,2,3-triazole and 1,5-dimethyl-1,2,3-triazole obtained by DFT calculations, as well as their excited (S_1_) and ground states (S_1_) predicted using time-dependent DFT (TDDFT) with a Gaussian’09 program (blue: N; grey: C; white: H), Table S1: TDDFT-predicted properties of models of 1,4-dimethyl 1,2,3-triazole and 1,5-dimethyl 1,2,3-triazole.
