# Supplementary material for: Synthesis of Dense 1,2,3-Triazole Polymers Soluble in Common Organic Solvents
Source: Polymers (Basel). 2021 May 17;13(10):1627. doi: 10.3390/polym13101627 (PMC8156623; doi:10.3390/polym13101627)
Supplement: Supplementary file 1 [file polymers-13-01627-s001.zip › polymers-1206885-supplementary.pdf]

polymers

Supplementary Materials: Synthesis of Dense 1,2,3-Triazole Polymers Soluble in Common Organic Solvents

Shota Yamasaki, Yuri Kamon, Linlin Xu, and Akihito Hashidzume

Table S1. TDDFT-predicted properties of models of 1,4-dimethyl 1,2,3-triazole and 1,5-dimethyl 1,2,3-triazole.

Model	Abs. [1]/ nm	Emission [2]/ nm
1,4-dimethyl-1,2,3-triazole	219.5	267.1
1,5-dimethyl-1,2,3-triazole	215.8	260.9

[1] Energy (TDDFT) using the optimized structure in ground state. [2] Optimization (TDDFT, root = 1) using the optimized structure in ground state.

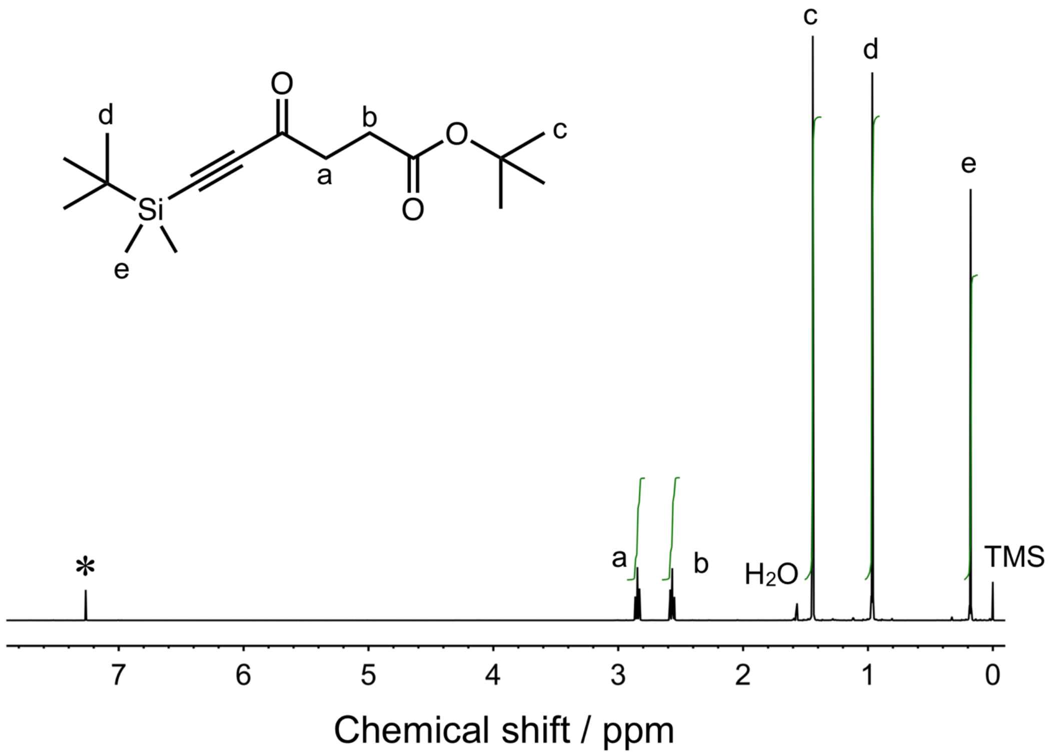

Figure S1. ^{1}H NMR spectrum for *t*-butyl 6-(*t*-butyldimethylsily)-4-oxo-5-hexynoate (**4**) ($CDCl_3$).

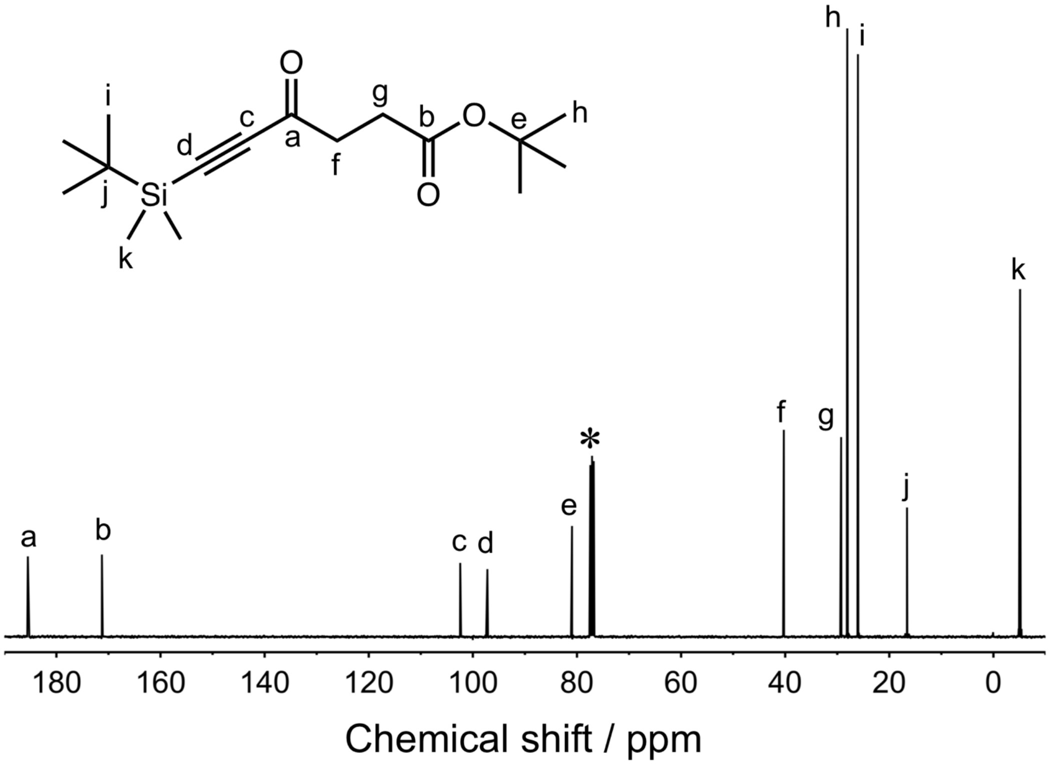

Figure S2. ^{13}C NMR spectrum for *t*-butyl 6-(*t*-butyldimethylsilyl)-4-oxo-5-hexynoate (**4**) ($CDCl_3$).

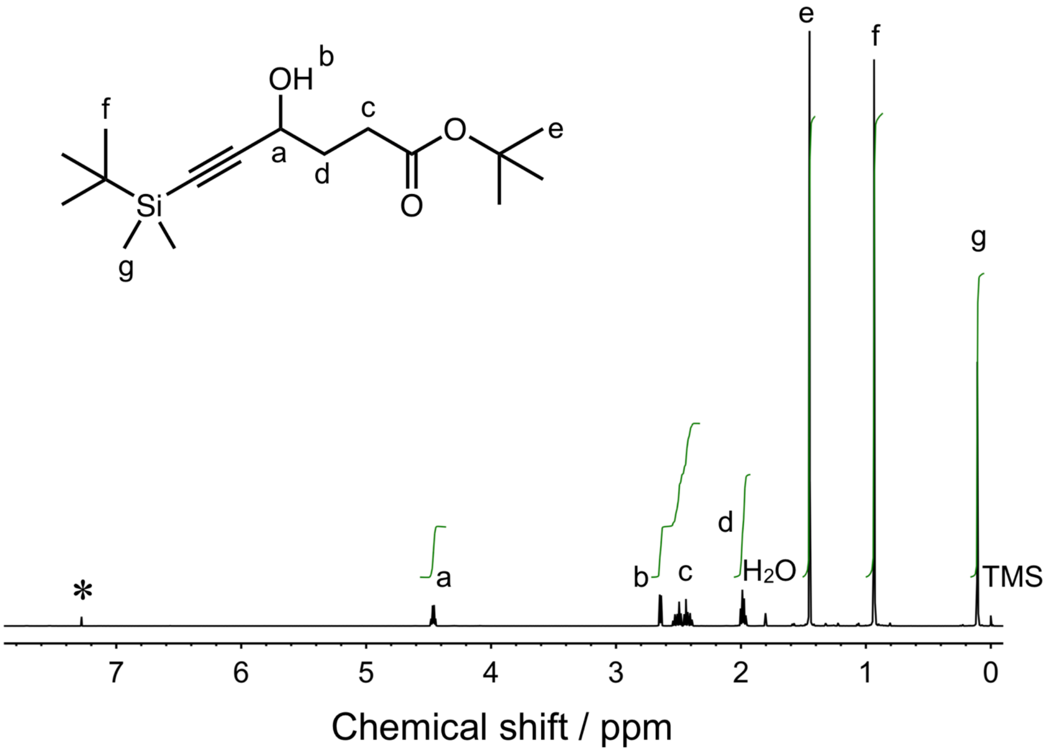

Figure S3. ^{1}H NMR spectrum for *t*-butyl 6-(*t*-butyldimethylsilyl)-4-hydroxy-5-hexynoate (**5**) ($CDCl_3$).

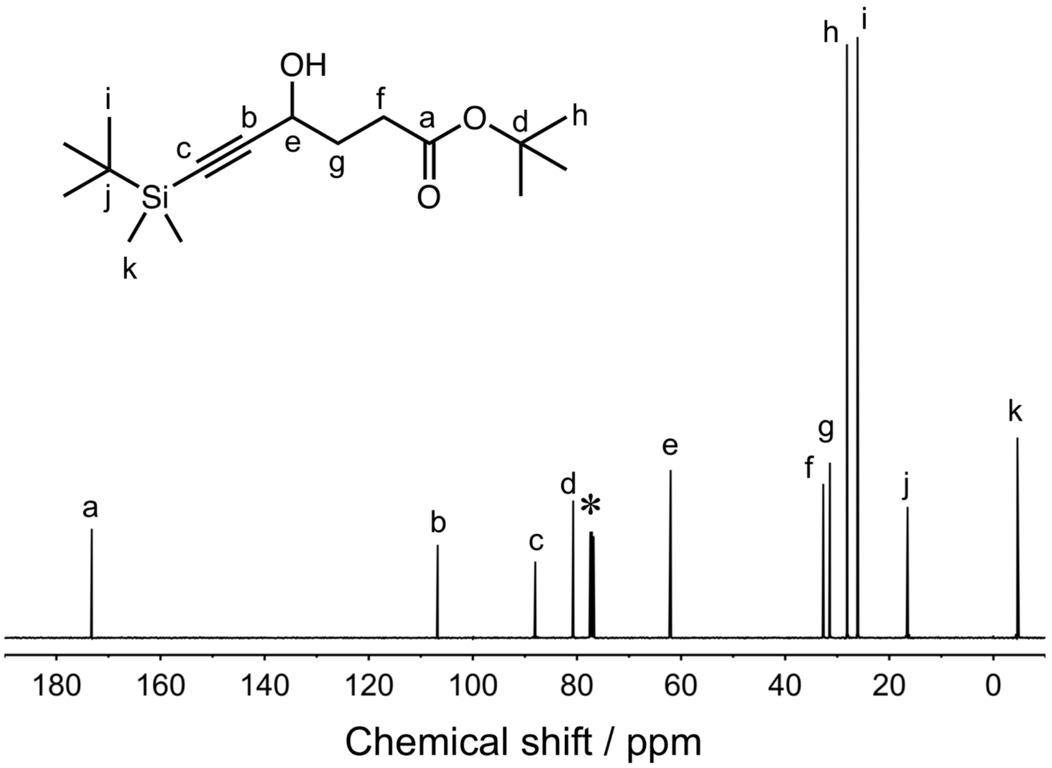

Figure S4. ^{13}C NMR spectrum for *t*-butyl 6-(*t*-butyldimethylsilyl)-4-hydroxy-5-hexynoate (**5**) ($CDCl_3$).

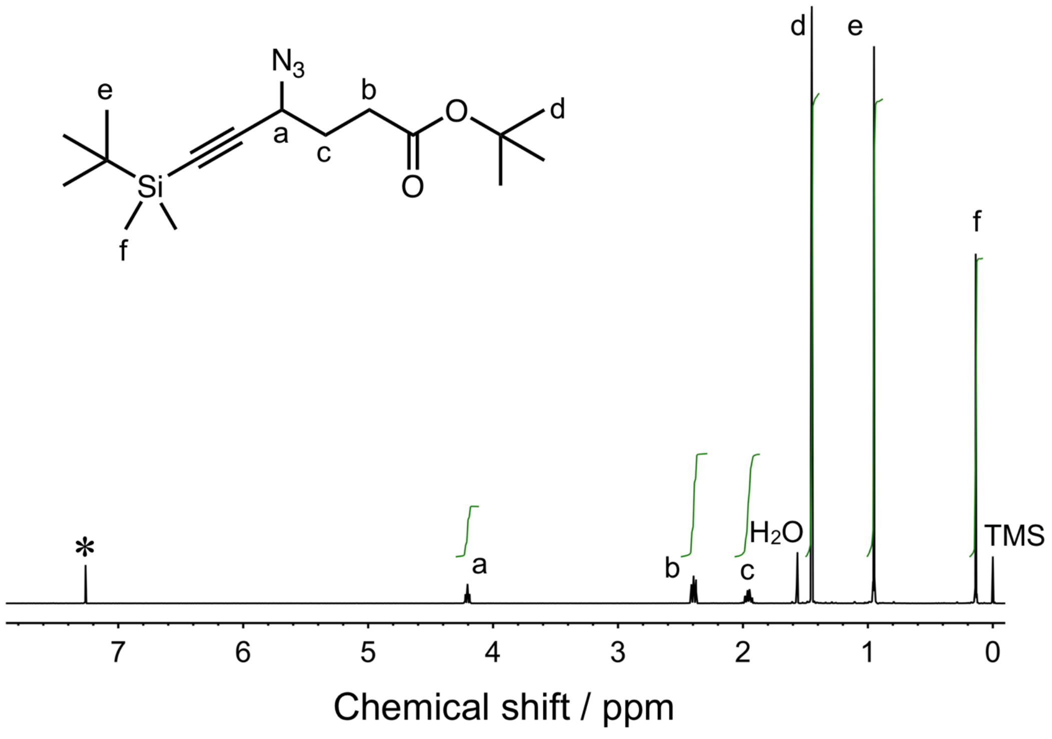

Figure S5. ^{1}H NMR spectrum for *t*-butyl 4-azido-6-(t-butyldimethylsilyl)-5-hexynoate (**6**) ($CDCl_3$).

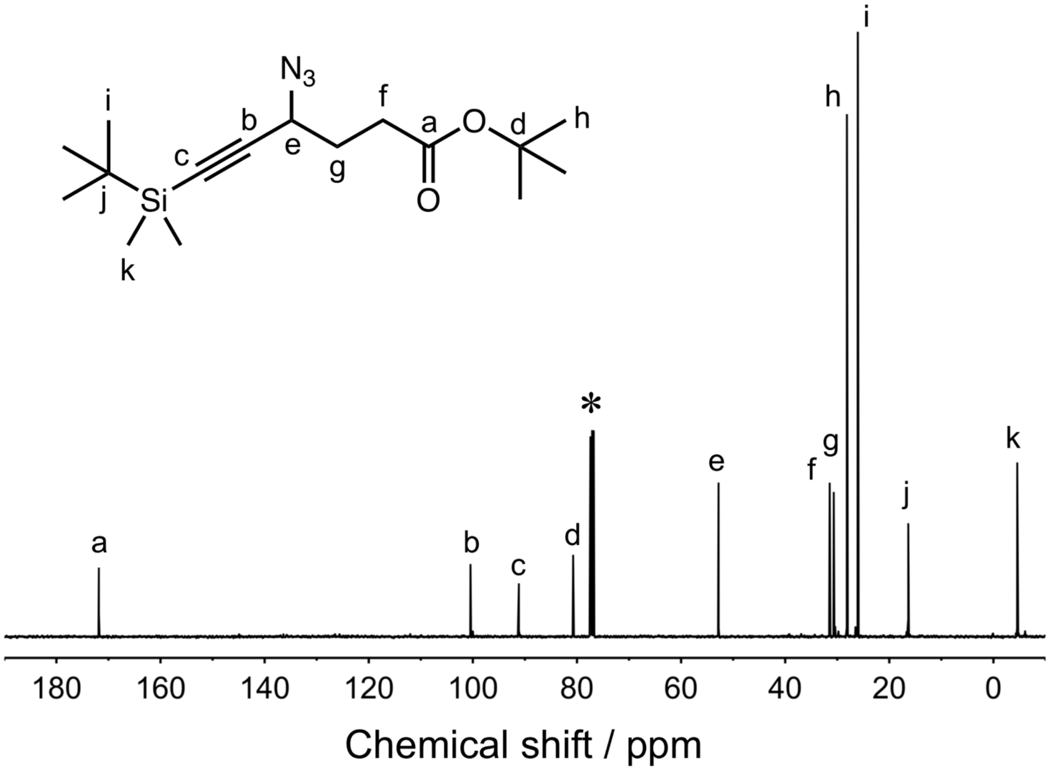

Figure S6. ^{13}C NMR spectrum for *t*-butyl 4-azido-6-(*t*-butyldimethylsilyl)-5-hexynoate (**6**) ($CDCl_3$).

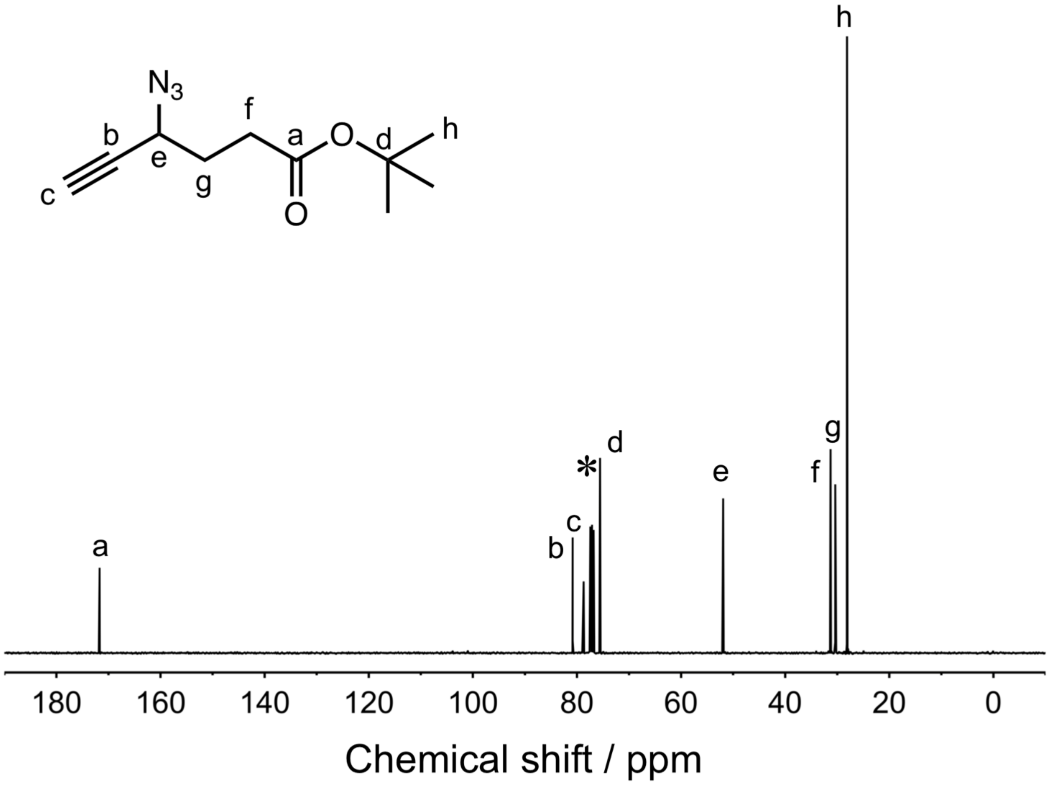

Figure S7. ^{13}C NMR spectrum for tBuAH ($CDCl_3$).

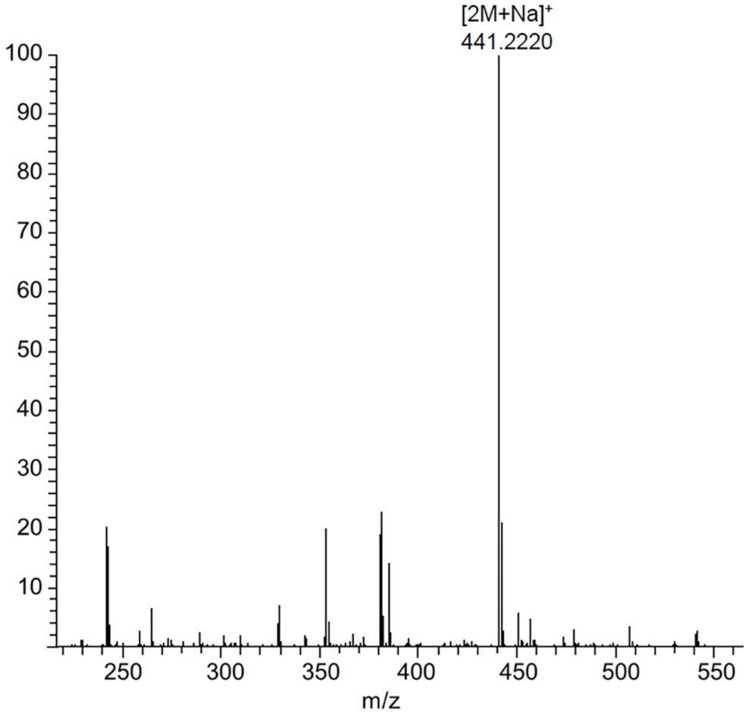

Figure S8. ESI-MS for tBuAH.

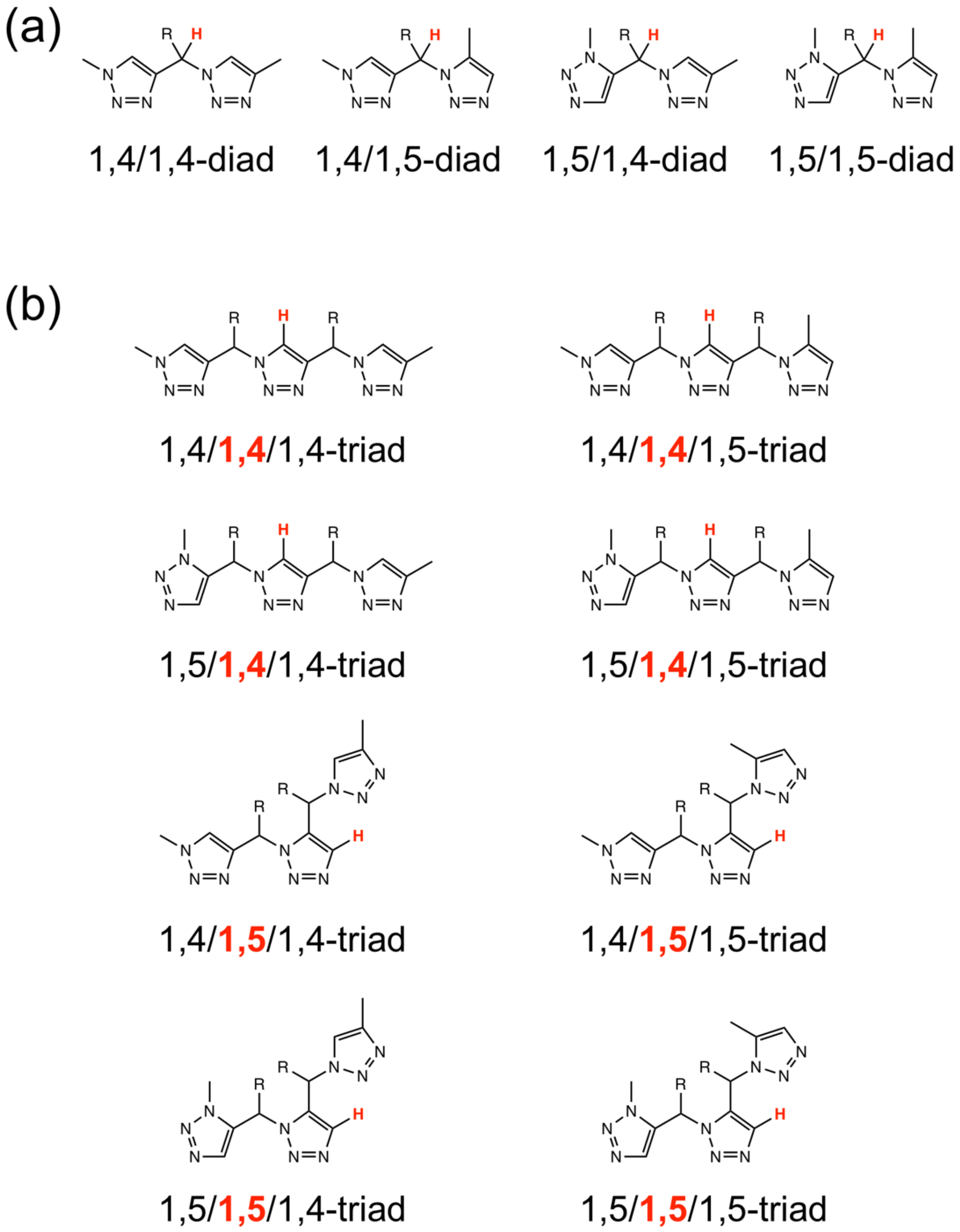

Figure S9. The structures of diads (a) and triads for poly(tBuAH) (b). The ^{1}H NMR signals due to the methine and triazole protons are separated based on the diads and triads, respectively.

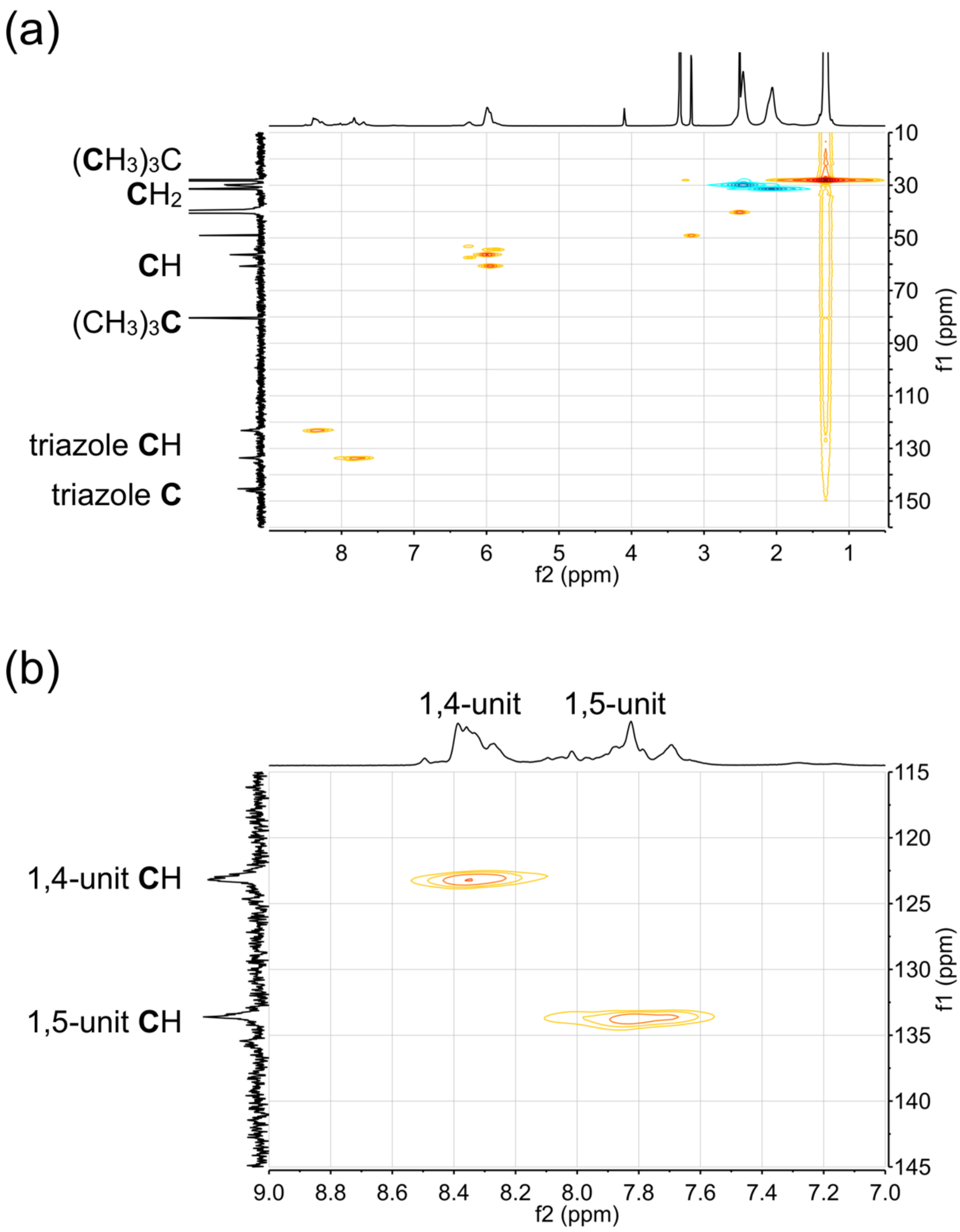

Figure S10. HSQC charts for poly(tBuAH) composed of 1,4- and 1,5-units (run 5); Whole (a) and expanded spectra (b).

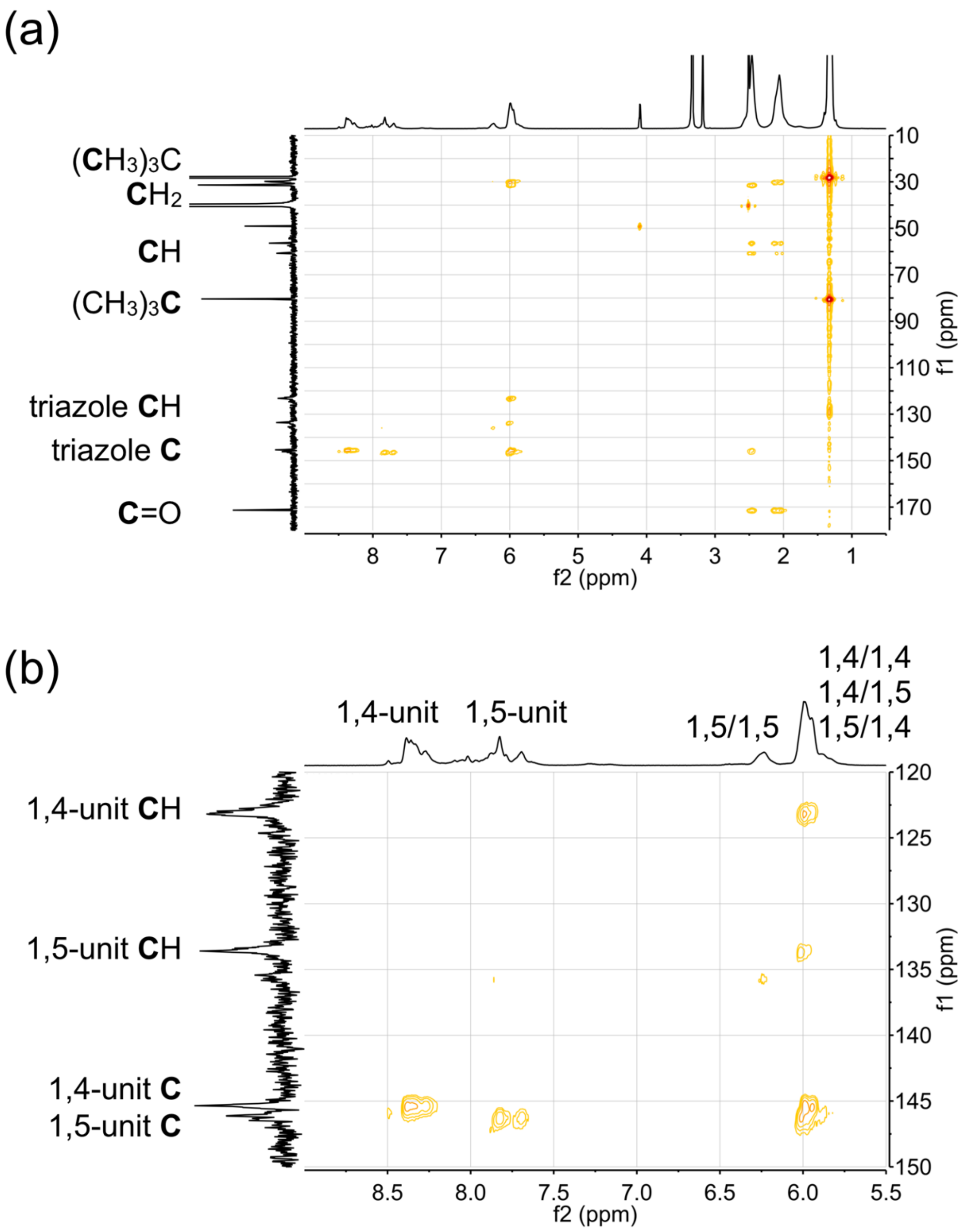

Figure S11. HMBC charts for poly(tBuAH) composed of 1,4- and 1,5-units (run 5); Whole (a) and expanded spectra (b).

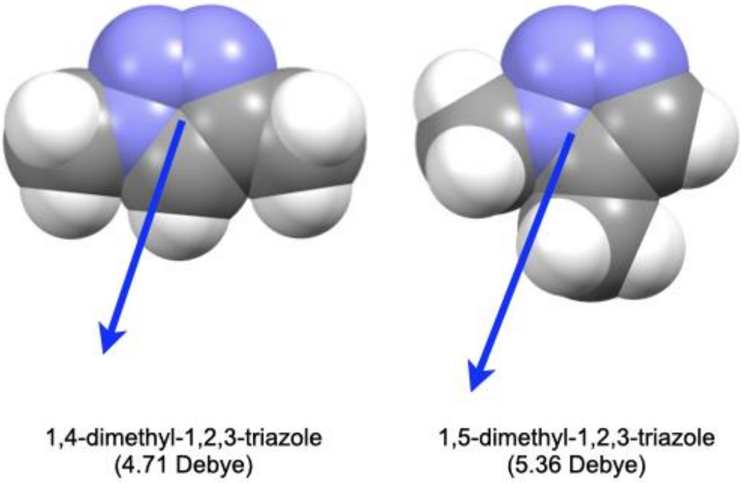

Figure S12. DFT calculation data for 1,4-dimethyl-1,2,3-triazole and 1,5-dimethyl-1,2,3-triazole.

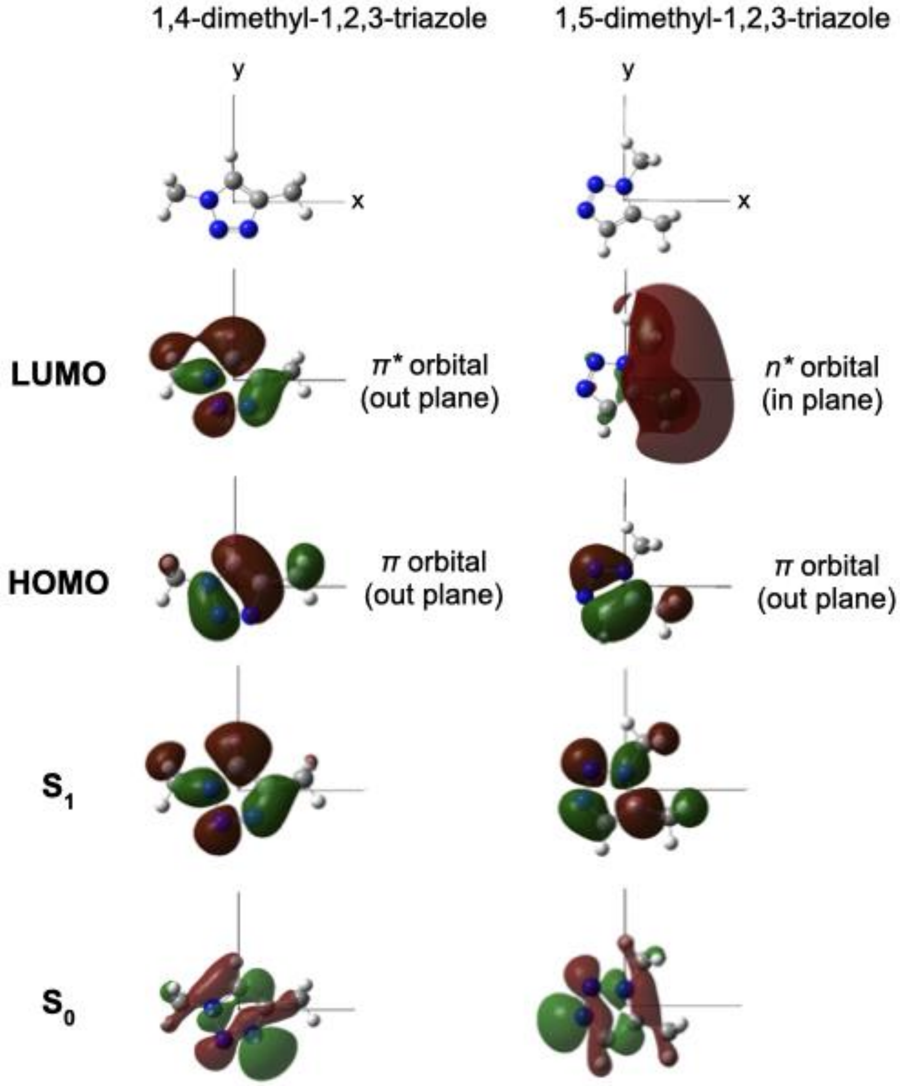

Figure S13. Optimized structures, the lowest-unoccupied molecular orbitals (LUMO), and the highest-occupied molecular orbitals (HOMO) of 1,4-dimethyl-1,2,3-triazole and 1,5-dimethyl-1,2,3-triazole obtained by DFT calculations, as well as their excited (S_1) and ground states (S_1) predicted by using TDDFT with a Gaussian'09 program (blue: N; grey: C; white: H).
